# Comparative Mitogenomes and Phylogenetic Analyses of Coccinellidae (Coleoptera: Coccinelloidea)

**DOI:** 10.1002/ece3.71053

**Published:** 2025-03-20

**Authors:** Qiaoqiao Liu, Pingzhou Zhu, Shiwen Xu, Chunyan Yang, Fan Song, Yufang Meng, Jinhong Zhou, Hailin Yang, Weidong Huang

**Affiliations:** ^1^ Department of Entomology and MOA Key Lab of Pest Monitoring and Green Management College of Plant Protection China Agricultural University Beijing China; ^2^ Genome Center of Biodiversity Kunming Institute of Zoology, Chinese Academy of Science Kunming China; ^3^ Yunnan Key Laboratory of Biodiversity Information Kunming China; ^4^ Yunnan International Joint Center of Urban Biodiversity Kunming China; ^5^ Yunnan Tobacco Company Yuxi China

**Keywords:** mitochondrial genome, noncoding region, phylogeny

## Abstract

Coccinellidae (ladybird beetles) comprises around 6900 described species with a worldwide distribution and exhibits a broad trophic diversity. Complete mitochondrial genomes (mitogenomes) are valuable resources in many research fields, such as genomics, population genetics, molecular evolution, and phylogenetics. Here we sequenced and report the complete mitogenome of *Calvia chinensis*, *Micraspis discolor* , *Harmonia eucharis*, and *Oenopia kirbyi*. By comparing with the 36 complete mitogenomes published in GenBank, we found that the long noncoding region (LNCR) between *trnI* and *trnQ* is present in the mitogenome of Chilocorini and Coccinellini, and the size of LNCR is positively correlated with their mitogenome size. The variable number tandem repeat (VNTR) was detected in the LNCR of *Calvia chinensis* and *Oenopia kirbyi*, indicating that the LNCR may be associated with the transcriptional regulation of the mitogenome. Heterogeneity in the base composition was encountered among the mitogenomes in Coccinellidae, especially in Noviini and some species of Epilachnini and Coccinellini, which may lead to unstable phylogenetic topologies. Phylogenetic relationships have been reconstructed by maximum likelihood and Bayesian inferences based on two mitogenomic datasets, PCG_rRNA (all 13 PCGs and two rRNAs) and PCG12_rRNA (all 13 PCGs with the third codon position excluded and two rRNAs). Our results are close to the subfamily and tribe classification system reported in previous studies and suggest the maximum likelihood analysis based on the PCG12_rRNA dataset is more sensitive in avoiding the false grouping of unrelated taxa with similar base composition in the reconstruction of the phylogeny.

## Graphical abstract

This study presents the complete mitochondrial genome (mitogenome) of four Coccinellidae species, containing Calvia chinensis, *Micraspis discolor* , Harmonia eucharis, and Oenopia kirbyi. Firstly, we reported the organization and nucleotide composition of these mitogenomes and compared them with those of other published mitogenomes of Coccinellidae. Then, the structures of the LNCR have been reported in detail. The evolutionary rate and nucleotide diversity of PCGs of Coccinellidae mitogenomes were calculated. Finally, the phylogeny of Coccinellidae was analyzed based on the two mitogenomic datasets using maximum likelihood and PhyloBayesian inference approaches.

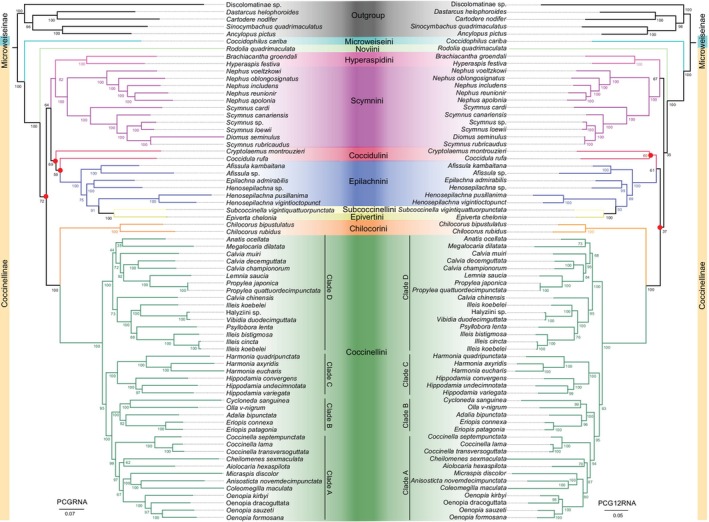

## 1 Introduction

The family Coccinellidae (ladybird beetles) comprises around 6900 described species belonging to 360 genera and 25 tribes (Robertson et al. 2015). Ladybird beetles have a worldwide distribution, but with most species recorded from tropical or subtropical regions (Robertson et al. 2015). It is widely known that this charismatic group includes many beneficial species that are voracious predators of the insects belonging to the hemipteran suborder Sternorrhyncha (aphids, scales, mealybugs, whiteflies, and psyllids) (Obrycki et al. 2009). Coccinellidae exhibits in fact a broad trophic diversity that while most coccinellids are predators, some are specialists on plant material, whereas others feed on fungi (Giorgi et al. 2009).

Our insight into the higher‐level phylogenetic relationships of ladybird beetles has benefited from the advances in sequencing technology; some recent molecular phylogenetic studies have changed the classification system of Coccinellidae, which early recognized six or seven subfamilies based on morphological features (Magro et al. 2010; Seago et al. 2011; Robertson et al. 2015; Che et al. 2021). Recently, (Che et al. 2021) conducted a phylogenomic analysis based on extensive sampling of species and genes and formally revised the subfamily classification of Coccinellidae to comprise three subfamilies: Microweiseinae, Monocoryninae, and Coccinellinae. However, this new view needs to be validated by more evidence, and the relationships among tribes have not yet been well resolved.

In the last decade, high‐throughput sequencing technologies and the rapid advance of bioinformatics instruments and statistical approaches have become a common source of nuclear and mitochondrial data for engaging in the study of nonmodel organismal systematics and phylogeography (Menezes et al. 2024). Due to the fact that mitochondrial DNA is present in multiple copies per cell, retrieving complete mitochondrial genomes (mitogenomes) from sequencing reads has become more convenient. Despite the genomics era, mitogenomes remain a widely utilized source of genetic data for animal phylogenetics, species delimitation, and demographic history (Guo et al. 2023; Huang, Zhang et al. 2023; Huang, Zhu et al. 2023). Several intrinsic traits of mitogenomes have been considered as suitability for these analyses, such as unambiguous orthologs, broadly uniform rates of molecular evolution, phylogenetic signals at diverse taxonomic scales, and uniparental inheritance consistent with bifurcating phylogenetic trees (Curole and Kocher 1999; Papadopoulou et al. 2010). The mitogenome of insects typically contains 13 protein‐coding genes (PCGs), 22 transfer RNAs (tRNAs), two ribosomal RNAs (rRNAs), and a putative control region (Cameron 2014). The whole mitogenome not only contains more sequence information than individual genes but also provides a suite of genome‐level characters, including gene rearrangement, strand asymmetry in nucleotide composition, and evolutionary patterns of the control region (Boore 1999; Cameron 2014).

The seven‐spotted ladybird beetle, *Coccinella septempunctata* (Linnaeus 1758) is the first species of ladybird that was sequenced for its complete mitogenome (Kim et al. 2012), which was determined to be 18,965 bp in length, and the control region, which is 4469 bp in length, is notable as the longest in coleopteran insects (Kim et al. 2012). Up to now, only 62 complete or nearly complete mitogenomes can be found in GenBank (data to Sep. 2024). Moreover, previous studies of coccinellid mitogenomes simply reported the length and some general characteristics, lacking comparative analyses (Kim et al. 2012; Zhang et al. 2023). Recently, mitogenomes have been used to infer phylogenetic relationships within Coccinellidae (Song et al. 2020; Iqbal et al. 2024), and the resulting topologies were consistent with the results of previous studies based on molecular and/or morphological data (Magro et al. 2010; Seago et al. 2011), highlighting the utility of mitogenomes in the phylogenetic study of Coccinellidae. However, the sequenced mitogenomes of many taxonomic categories within Coccinellidae remain poorly represented or unrepresented. This situation impedes our understanding of the mitogenomic evolution of Coccinellidae and using mitogenomic sequences as alternative evidence to reinforce building a natural classification within Coccinellidae.

In this study, we determined the complete mitogenome of *Micraspis discolor* (Fabricius 1798), *Harmonia eucharis* (Mulsant 1853), *Calvia chinensis* (Mulsant 1850) and *Oenopia kirbyi* Mulsant, 1850 using next‐generation sequencing to gain insight into the mitogenome characteristics and phylogeny of ladybird beetles. The genomic structure, base composition, AT content, and evolutionary rates of these mitogenomes were investigated. Subsequently, combined with other mitogenomes downloaded from GenBank, we conducted comparative analyses to explore the variability and evolutionary rate of mitogenomes within the Coccinellidae. Finally, phylogenetic relationships within Coccinellidae were inferred using both maximum likelihood and Bayesian inference methods based on the mitogenomes.

## 2 Materials and Methods

### 2.1 Sample and DNA Extraction

Adult specimens of *C. chinensis* , *H. eucharis* , *M. discolor* , and *O. kirbyi* were collected from tobacco fields in Guizhou and Yunnan Provinces, China (Table S1). Living specimens were soaked in absolute ethyl alcohol after being captured in the field and stored at −20°C prior to DNA extraction. Specimens were identified based on their morphological features. Furthermore, *H. eucharis* and *M. discolor* were also confirmed by nucleotide BLAST with the sequences numbered KP829575 and MT762085 in the NCBI database. Total genomic DNA was extracted from the prothoracic muscle by the DNeasy Blood and Tissue kit (Tiangen, Beijing, China) following the manufacturer's instructions. The remaining tissue and DNA evidence were preserved under −20°C in the Entomological Museum of China Agricultural University (Beijing China).

### 2.2 DNA Sequencing, Assembly, and Annotation

The 350 bp small fragment libraries were constructed for each species and then sequenced using the Illumina NovaSeq 6000 platform with 150 bp paired‐end reads. The adapters were trimmed from raw reads using Trimmomatic (Bolger et al. 2014). Remaining high‐quality reads were used to perform a *de novo* assembly by IDBA‐UD (Peng et al. 2012) with minimum and maximum *k* values of 45 bp and 145 bp, respectively. Subsequently, clean reads were mapped onto the obtained mitogenome sequences using Geneious (Kearse et al. 2012) to confirm the accuracy and integrity of the assembled mitogenomes, with mismatches of up to 2%, a maximum gap size of 3 bp, and a minimum overlap of 40 bp. MitoZ (Meng et al. 2019) was used to annotate the mitochondrial encoding genes preliminarily. The gene boundary of tRNA was further confirmed by tRNAscan‐SE (Lowe and Eddy 1997). Finally, the gene boundaries of PCG and rRNA genes were further corrected by aligning with homologous genes of previously sequenced ladybird beetle mitogenomes.

### 2.3 Sequence Analysis

MEGA 7 (Kumar et al. 2016) was used to calculate the nucleotide composition of sequences and the synonymous codon usage (RSCU) of PCGs. The AT‐skew and GC‐skew of four newly sequenced mitogenomes were calculated according to the formulae (A − T)/(A + T) and (G − C)/(G + C), respectively. Then, DnaSP 6.0 (Rozas et al. 2017) was used to estimate nucleotide diversity (Pi) with a sliding window analysis (a sliding window of 100 bp and a step size of 20 bp). The nonsynonymous (Ka)/synonymous (Ks) substitution rates among the 13 PCGs of Coccinellidae were also calculated by DnaSP 6.0. We used Tandem Repeats Finder (Benson 1999) to determine tandem repeat units of the noncoding region with the default parameters. Finally, circular mitogenome maps were generated by Geneious prime (Kearse et al. 2012).

In the process of mitogenome annotation, we observed a long noncoding region (LNCR) between the *trnI* and *trnQ* genes in Chilocorini and Coccinellini. To examine correlations between the size of LNCR and mitogenomic size in ladybird beetles, we conducted the Pearson's correlation test. Additionally, the correlations between the size of the control region and mitogenomic size also were investigated. These statistical analyses were performed in Origin 2024b (OriginLab Corporation, Northampton, MA, USA).

### 2.4 Sequence Heterogeneity and Phylogenetic Analysis

To investigate the phylogenetic relationships of Coccinellidae, we have constructed the most complete mitogenome dataset of ladybirds to date. A total of 71 mitogenomes were analyzed in this study, including our four newly determined mitogenomes and 62 mitogenomes of ladybird beetles downloaded from the NCBI database. In addition, five other species of Coleoptera were used as outgroups to root the tree (Table S2).

Sequence of each PCG was individually aligned using MAFFT implemented in TranslatorX (Abascal et al. 2010) online platform following the invertebrate mitochondrial genetic code and removed the poorly aligned sites by GBlocks with default settings (Talavera and Castresana 2007). Sequence of the rRNA gene was aligned using the MAFFT v7.0 online server with G‐INS‐i strategy (Katoh et al. 2019) and ambiguous positions in the alignment were also filtered using GBlocks (Talavera and Castresana 2007). The third codon position of the PCG alignments was excluded in MEGA 7 (Kumar et al. 2016). Then, aligned sequences were used to concatenate the PCG_rRNA (10,908 bp in total, included all 13 PCGs and two rRNAs) and PCG12_rRNA (9175 bp in total, included all 13 PCGs with the third codon position excluded and two rRNAs) datasets by Geneious prime for further phylogenetic analysis. The heterogeneity divergence within the PCG datasets was analyzed using AliGROOVE (Kück et al. 2014) with the default sliding window size.

Both maximum likelihood (ML) analysis and Bayesian inference (BI) were used to reconstruct the phylogenetic tree based on the two datasets, PCG_rRNA and PCG12_rRNA. The ML tree was constructed using an ultrafast bootstrap method with 1000 replicates in the IQ‐TREE web server based on the unpartitioned dataset (Trifinopoulos et al. 2016). According to the Bayesian Information Criterion (BIC), GTR + F + I + G4 is selected as the best‐fit model. The BI tree was constructed by PhyloBayes 1.8c (Lartillot et al. 2013) on the CIPRES Science Gateway (Miller et al. 2010). In each BI analysis, a site‐heterogeneous mixture model (CAT + GTR) with a discrete gamma distribution of 4 was selected to avoid the false grouping of unrelated taxa with similar base composition and accelerated evolutionary rates. Two independent Markov Chain Monte Carlo chains of 5000 generations each were performed. Convergence was evaluated with the “bpcomp” and “tracecomp” programs in the PhyloBayes package by calculating the largest (maxidiff) and mean (meandiff) discrepancies observed across all partitions. BI runs are considered to have achieved satisfying convergence when the maxdiff was less than 0.1. A consensus tree was computed using the remaining trees from two runs after the initial 25% of trees were discarded as “burn‐in.”

## 3 Results and Discussion

### 3.1 Mitogenome Organization and Nucleotide Composition

**FIGURE 1:**
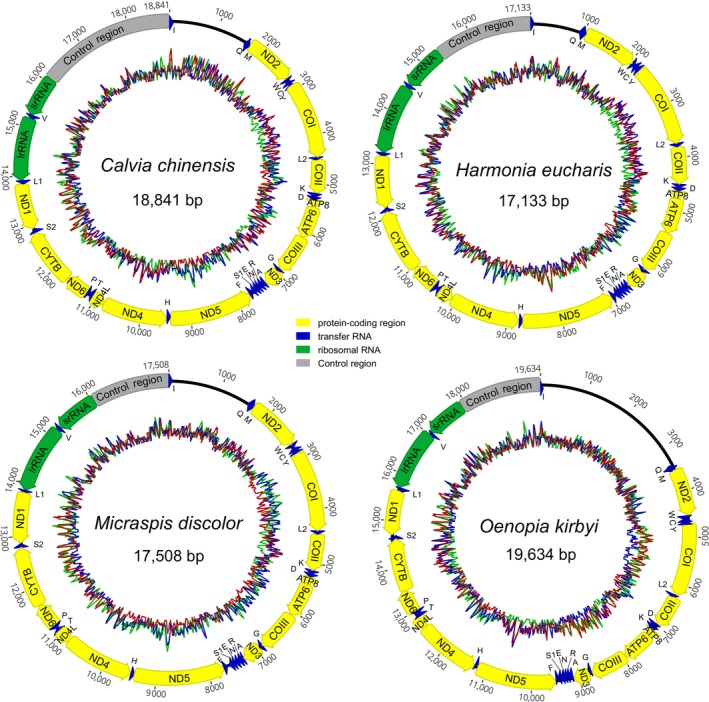
Mitogenomic maps of the four newly sequenced mitogenomes of Coccinellidae. Protein‐coding, ribosomal, and transfer RNA genes are indicated with standard abbreviations. Gene orientations are indicated by arrow directions. The circular wavy lines show the frame plot.

The mitogenomes of *C. chinensis* (18,841 bp), *H. eucharis* (17,133 bp), *M. discolor* (17,508 bp), and *O. kirbyi* (19,634 bp) were sequenced completely as a single, circular double‐stranded DNA molecule (Figure 1). The mitogenomic lengths of *C. chinensis* , *H. eucharis* , and *M. discolor* are medium‐sized compared with the other 36 complete Coccinellidae mitogenomes analyzed in this study, which ranged from 15,343 bp (*Coccidophilus cariba*) (Nattier and Salazar 2019) to 19,413 bp ( *Coccinella septempunctata* ), while *O. kirbyi* is the largest mitogenome in Coccinellidae so far. The newly sequenced mitogenomes comprised 37 typical genes (13 PCGs, two rRNAs and 22 tRNAs) like other complete mitogenomes of ladybird beetles and insects (Boore 1999; Guo et al. 2023). These genes exhibit a typical arrangement like the majority bilaterian animals (Cameron 2014) and other ladybirds (Song et al. 2020), with 23 genes (nine PCGs and 14 tRNAs) encoded by the majority‐strand (J‐strand) and 14 genes (four PCGs, eight tRNAs and two rRNAs) encoded by the minority‐strand (N‐strand).

**FIGURE 2:**
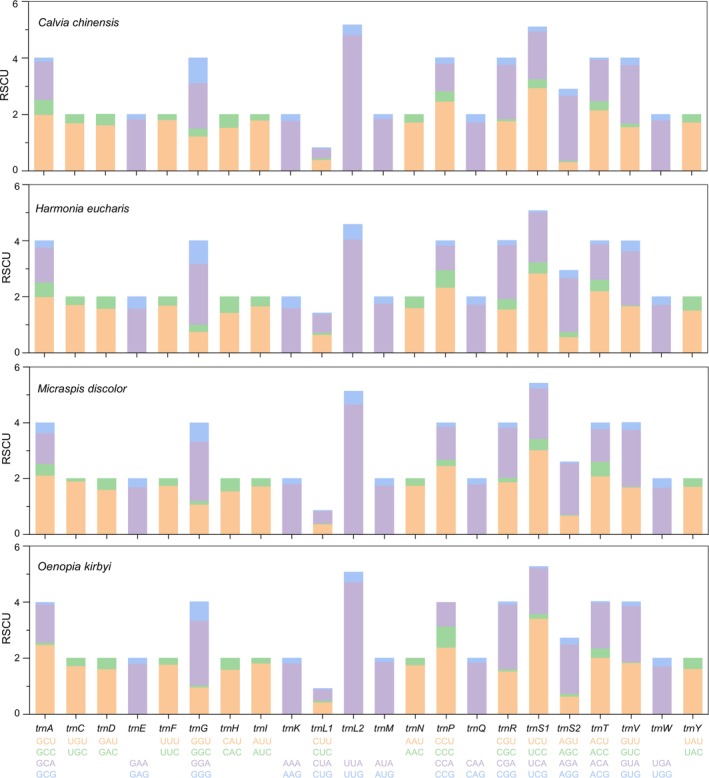
Relative synonymous codon usage (RSCU) in four newly sequenced mitogenomes.

In the mitogenomes of Coccinellidae, a typical start codon of ATN (ATA/ATT/ATG/ATC) was detected in the majority of PCGs, especially for *Cycloneda sanguinea* with ATG at the initiation of all 13 PCGs. But in some cases, the start codon of *ATP6*, *COI*, *COII*, *ND1*, *ND2*, and *ND3* was TTG. In this study, ATN was detected to initiate the PCGs of the newly sequenced mitogenomes, except the *COI* of *H. eucharis* and *M. discolor* , which roughly located around the 5′ end of *trnY* and lacks the start codon like *Aiolocaria hexaspilota* and *Hippodamia variegata* (Hao et al. 2019; Seo et al. 2019). The 5′ end of *COI* is highly variable in the Coccinellidae mitogenomes. Unlike the start codon, which is usually complete, the complete stop codons TAA or TAG were only detected in *ATP8*, *ND1*, and *ND4* of the four mitogenomes, and the *ATP6* of *M. discolor* and *O. kirbyi*. The incomplete stop codon TA was detected in the *CYTB* of *O. kirbyi*, and a single T was detected in the remaining PCGs. Incomplete stop codons are prevalent in other insect mitogenomes (Guo et al. 2023; Huang, Zhang et al. 2023) and will recover to the complete by a posttranscriptional polyadenylation (Li et al. 2009, Liu et al. 2024). The relative synonymous codon usage (RSCU) analysis of the four mitogenomes showed a strong bias toward A and T, especially the third codon position of the amino acids (Figure 2). The most frequently encoded amino acids were *trnS1*, *trnL2*, *trnA*, *trnG*, *trnP*, *trnR*, *trnT*, and *trnV*, which had the highest RSCU values. The most commonly used codons, UUA, AUU, UUU, and AUA, comprised solely A or T, which reflected the high AT content of PCGs.

Twenty‐two typical tRNA genes were identified in the four ladybird mitogenomes, with lengths ranging from 56 to 70 bp in *C. chinensis* , 56**–**71 bp in *H. eucharis* , 54**–**70 bp in *M. discolor* , and 56**–**69 bp in *O. kirbyi* (Tables S3–S6). All tRNAs could be folded into a typical structure like cloverleaf, except for several exceptions. The *trnS1* lacks the dihydrouridine (DHU) and *trnP* lacks the TψC arm, forming a simple loop (Figure S1) as in many ladybirds and metazoan mitogenomes (Huang, Zhang et al. 2023). In addition, the TψC arm of *trnD* in *H. eucharis* and *M. discolor* was replaced with a large loop due to unmatched base pairs (Figure S1), which was reported first in Coccinellidae. Two rRNA genes were encoded on the N‐strand in the four newly sequenced mitogenomes. The *lrRNA* was located between *trnL1* and *trnV*, ranging from 1278 bp to 1299 bp, whereas the *srRNA* was located between *trnV* and CR, ranging from 807 bp to 829 bp (Figure 1, Tables S3–S6).

The average AT content of the four mitogenomes is similar, with 79.03% in *C. chinensis* , 76.10% in *H. eucharis* , 78.79% in *M. discolor* , and 79.05% in *O. kirbyi*, reflecting the strong AT bias, of which the CR was the highest among the components of the mitogenome (Table S7), similar to that of other Coccinellidae species (Salazar and Nattier 2020; Li et al. 2021; Huang, Zhu et al. 2023; Zhang et al. 2023). In addition, the PCGs, rRNAs, and tRNAs were all biased in nucleotide composition, with AT content greater than twice the GC content. Furthermore, the mitogenomes exhibit positive AT‐skews (0.026–0.056) and negative GC‐skews (−0.126 to −0.227), which is also common for ladybird beetles and Coleoptera (Guo et al. 2023; Huang, Zhu et al. 2023; Iqbal et al. 2024).

### 3.2 Noncoding Region

**FIGURE 3:**
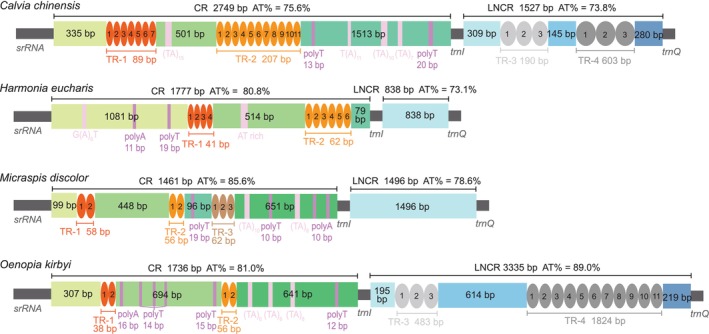
Organization of the control region and long noncoding region in four newly sequenced mitogenomes. The location and copy number of tandem repeats were shown by colored ovals with Arabic numerals inside. The remaining sequences of the control region were indicated by the colored rectangle. CR: Control region; LNCR: Long noncoding region; TR: Tandem repeats.

In the four newly sequenced Coccinellidae mitogenomes, majority of genes are arranged compactly with few intergenic spacers ranging from 1 bp to 53 bp (Tables S3–S6). In addition, each mitogenome contains two large noncoding regions located between *srRNA‐trnI* and *trnI‐trnQ*. The length of the noncoding region between *srRNA* and *trnI* is 2749 bp in *C. chinensis* , 1777 bp in *H. eucharis* , 1461 bp in *M. discolor* , and 1736 bp in *O. kirbyi*, while the length between *trnI* and *trnQ* is 1527 bp in *C. chinensis* , 838 bp in *H. eucharis* , 1496 bp in *M. discolor* , and 3335 bp in *O. kirbyi*, respectively. The longest noncoding region in majority of insect mitogenomes is CR and is located between *srRNA* and *trnI* as usual (Cameron 2014; Hu et al. 2019). However, in *M. discolor* and *O. kirbyi*, the longest noncoding region is located between *trnI* and *trnQ*. To ensure the location of CR in these four mitogenomes, we investigated the components of these noncoding regions. The results showed that the noncoding region between *srRNA‐trnI* contains a variable number of tandem repeats (VNTRs) and the motifs (polyT/polyA, (TA)n, G(A)nT and T(A)n) (Figure 3), which have been recognized to regulate the duplication and transcription of the mitogenome (Liang et al. 2022; Liu et al. 2024), indicating that the CR of these mitogenomes is located between *srRNA* and *trnI* like other ladybirds and majority of insects (Cameron 2014; Song et al. 2020). However, the long noncoding region between *trnI* and *trnQ* (hereinafter referred to as “LNCR”) does not contain motifs, and the VNTRs were only detected in *C. chinensis* and *O. kirbyi*. In general, the VNTRs of CRs are much smaller than those of LNCRs, and the extension of LNCR is mainly due to the tandem repeats.

**FIGURE 4:**
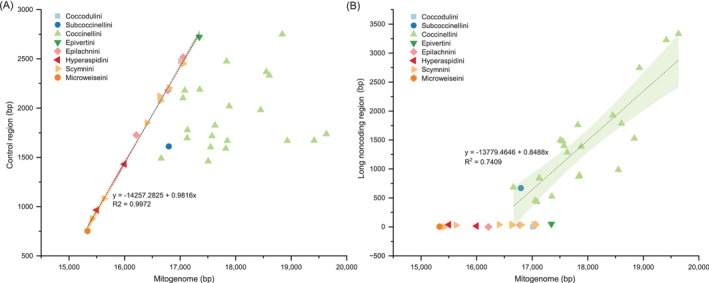
Pearson correlation of length between size of control region (A) or long noncoding region (between *trnI* and *trnQ*) (B) and mitogenome size in Coccinellidae.

The length of CR is considered to be the most important factor affecting the size of insect mitogenomes (Hu et al. 2019; Tyagi et al. 2020; Zhang et al. 2021). Among the 40 coccinellid complete mitogenomes (range from 15,335 bp to 19,634 bp), the size of CR and LNCR ranges from 753 bp to 3124 bp and from 0 bp to 3335 bp, respectively. Correlation analyses exhibited that the Coccinellidae mitogenome size is strongly correlated with the length of LNCR (Pearson's correlation coefficient = 0.84224), but weakly correlated with the length of CR (Pearson's correlation coefficient = 0.43801). Further investigation found that the genetic spacer between *trnI* and *trnQ* (less than 51 bp) is far less than its CR length (from 753 bp to 3124 bp) in the mitogenomes of Coccidulini, Epivertini, Epilachnini, Hyperaspidini, Scymnini, and Microweiseini. In these mitogenomes, the variation of mitogenome size is significantly attributed to the length of CR (Figure 4A), which is similar to other insects (Guo et al. 2023; Huang, Zhang et al. 2023; Huang, Zhu et al. 2023). However, the length of LNCR between *trnI* and *trnQ* in the mitogenomes of Coccinellini and Chilocorini ranges from 433 bp to 3335 bp, which is much larger than the CR (range from 1461 bp to 2749 bp) sometimes. In these two tribes, the length variation of the mitogenomes is attributed to LNCR (Figure 4B) instead of CR.

In the majority insect mitogenomes, the mitochondrial gene is arranged compactly with no introns and with few intergenic nucleotides, and neighboring genes may even shortly overlap in some cases, with CR being the largest noncoding region (Li et al. 2009, Cameron 2014). The LNCR of mitogenomes appears to be the opposite of the compactness principle. Recently, all the long noncoding regions have been identified as CRs in a few mitogenomes of Thysanoptera and Hymenoptera. The CRs of *Frankliniella*, *Thrips*, and *Sericothripis* in Thysanoptera are easy to identify with duplicated sequences among the noncoding regions (Shao and Barker 2003; Yan et al. 2012, 2014; Chakraborty et al. 2018), and further investigation in the transcriptome of *Sericothripis houjii* confirmed the duplicated CRs are functional in the transcription of the mitogenome (Liu et al. 2024). In *Aphidius gifuensis*, the LNCR between *trnM* and *trnQ* has no repeats compared with the CR between *srRNA* and *trnI* but contains the transcription initiation site of the N‐strand (Zhao et al. 2023). The CR and LNCR structure of the coccinellid mitogenome, which is represented by the four newly sequenced mitogenomes, is similar to that of *Aphidius gifuensis*. Although no repeat sequence was detected in the LNCR when compared with their CR, the VNTRs were detected in *C. chinensis* and *O. kirby*i (Figure 4). Given the fact that VNTR has been found to be strongly associated with the expression of the proximal genes (Bakhtiari et al. 2021), the LNCR of *C. chinensis* and *O. kirby* may be another CR of the mitogenome. However, whether the LNCR is functional in the transcriptional regulation of coccinellid mitogenomes needs more research about their transcriptome.

### 3.3 Evolutionary Rate and Nucleotide Diversity in the PCGs of Coccinellid Mitogenomes

**FIGURE 5:**
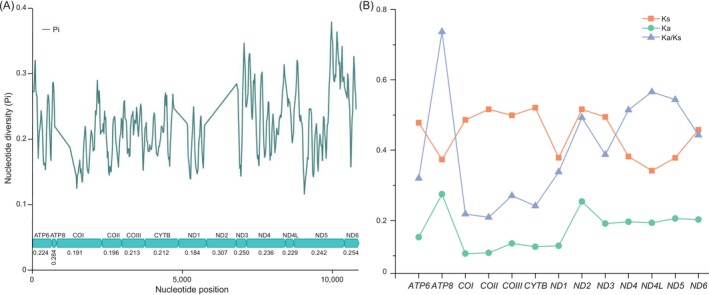
(A) Nucleotide diversity of 13 PCGs among 66 Coccinellidae mitogenomes. The green line shows the value of nucleotide diversity (Pi). The Pi value for each gene is shown in the graph. (B) Evolutionary rate of 13 PCGs. Ks is the synonymous substitution rate, Ka is the nonsynonymous substitution rate, and Ka/Ks is the ratio of nonsynonymous substitution/synonymous substitution.

By sliding window analysis, the 13 PCGs of 66 Coccinellidae mitogenomes exhibited a high variation of nucleotides (Figure 5A). The nucleotide diversity value is ranged from 0.184 (*ND1*) to 0.307 (*ND2*). Of which, *ND2* presented the highest variability, followed by *ATP8* (Pi = 0.284) and *ND6* (Pi = 0.254). However, *ND1* (Pi = 0.184) and *COI* (Pi = 0.191) with relatively lowest nucleotide diversity, indicated that they were comparatively more conserved than other PCGs. Nucleotide diversity is a parameter guideline to identify the species‐specific markers. As the genetic diversity is much higher than the nuclear genes (Antil et al. 2022), mitochondrial genes have been used to bioassay. Especially, a 658 bp sequence of the *COI* gene is most widely used in species‐level identification among insects, which is known as DNA barcoding (Schmidt et al. 2015; Yi et al. 2018; Jiménez‐García et al. 2023). The previous study showed that the interspecific divergences of the ladybirds DNA barcoding ranged from 10% to 29.1% (Huang et al. 2020). However, the nucleotide diversity analysis in this study exhibited that the genetic diversity of *COI* (Pi = 0.191) is comparatively lower among the PCGs of the Coccinellidae mitogenome, indicating that the other PCGs contain more information about population genetics and species delimitation in Coccinellidae, such as *ND2*, *ND3*, and *ND6*, all of which exhibited sufficient nucleotide diversity (Pi > 0.250).

Both the synonymous and nonsynonymous substitution was suggested to contribute to evolution (Bailey et al. 2021; Jiang et al. 2023). To further analyze the evolutionary rate of the Coccinellidae mitogenomes, the synonymous substitution rate (Ks) and nonsynonymous substitution rate (Ka) were calculated in each PCG of 66 ladybirds mitogenomes, and the Ka/Ks value was further calculated to estimate the selective pressure (Hurst 2002). Our results showed that the Ka/Ks value of each PCG was less than 1 (Figure 5B), indicating that all PCGs underwent purifying selection pressures. Among the 13 PCGs, *COII* (Ka/Ks = 0.209) and *COI* (Ka/Ks = 0.219) underwent comparatively stronger purifying selection and exhibited a lower evolutionary rate. In contrast, the Ka/Ks value of *ATP8* (0.737) was obviously higher than that of other PCGs (0.209–0.566), indicating that *ATP8* underwent the weakest purifying selection pressure among the PCGs. In general, cytochrome oxidase c genes underwent stronger purifying selection and a lower evolutionary rate than NADH dehydrogenase genes (Huang, Zhang et al. 2023; Huang, Zhu et al. 2023).

### 3.4 Sequence Heterogeneity and Phylogenetic Analysis

The heterogeneity of sequence variation was assessed using AliGROOVE separately for the datasets of PCG_rRNA (mean similarity score is 0.572) was higher than that of PCG12_rRNA (mean similarity score is 0.610). Results indicated that the third codon positions exhibited higher heterogeneity (mean similarity score is 0.142) than the first (mean similarity score is 0.590) and the second codon positions (mean similarity score is 0.798). Noviini exhibited notably higher heterogeneity than other tribes (Figures S2, S3) that may not be robustly placed or may be misplaced on phylogenetic trees (Guo et al. 2023). Some species of Epilachnini and Coccinellini exhibited relatively higher heterogeneity than other species within the same tribe, indicating that the inner phylogenetic relationships of these tribes may robustly recover or may be reconstructed inaccurately. A significantly higher heterogeneity was usually observed in the incomplete mitogenomes in our study, such as the mitogenome of *Coccidula rufa* and *Chilocorus bipustulatus* , which lack *ND2* and rRNAs, and rRNAs, respectively. The complete mitogenome may reduce the heterogeneity and be more helpful in reconstructing the phylogenetic tree into a correct topology, as suggested in a previous study (Guo et al. 2023).

**FIGURE 6:**
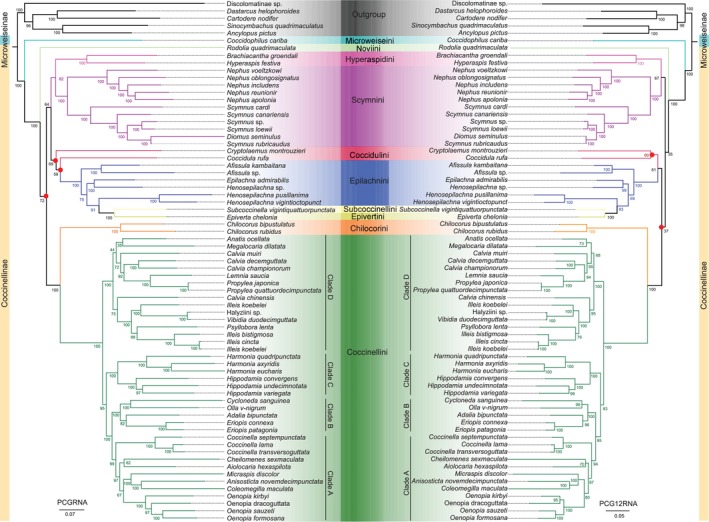
Phylogenetic tree of Coccinellidae obtained from maximum likelihood analyses based on the datasets of PCGRNA (left) and PCG12RNA (right). Different tribes are highlighted by colors. The red spot in the node of tree indicated the dispute between two trees. Bootstrap support values are shown near the nodes.

We found the phylogenies inferred with different datasets are similar (Figure 6), whereas different phylogenetic analysis methods have an important influence on our phylogenetic inferences (Figures S4). In ML trees, the group of Coccidulini, Epilachnini, Subcoccinellini, and Epivertini is sister to Hyperaspidini + Scymnini, supported by the PCG_rRNA dataset, but is sister to Chilocorini + Coccinellini by the PCG12_rRNA dataset. In PB trees, Hyperaspidini is grouped with Coccidulini, Epilachnini, Subcoccinellini, and Epivertini with a messy relationship instead of being sister to Scymnini in ML trees, and the inner relationships of Epilachnini and Coccinellini are disputed. It seems that BI cannot distinguish these groups well and reconstruct a stable phylogenetic relationship. Therefore, ML analysis seems to perform better than BI in avoiding the false grouping of unrelated taxa with similar base composition in the reconstruction of the phylogeny of Coccinellidae. In general, Microweiseini, represented by *Coccidophilus cariba*, recovered as sister to the rest of Coccinellidae in all our analyses. This finding received a high support value (BS = 100, PP = 1) in both the ML and BI analyses and is consistent with those of previous studies (Seago et al. 2011; Robertson et al. 2015; Che et al. 2021; Nattier et al. 2021; Tomaszewska et al. 2021).

The monophyly of Coccinellini is strongly supported (BS =100, PP = 1) in our trees regardless of tree‐building methods and datasets, which is in line with previous phylogenetic studies of ladybirds (Seago et al. 2011; Robertson et al. 2015; Escalona et al. 2017; Che et al. 2021; Nattier et al. 2021; Tomaszewska et al. 2021). Within Coccinellini, a mycophagous clade comprised *Halyzia*, *Vibidia*, *Psyllobora*, and *Illeis* also recovered as monophyletic with high support (BS = 100, PP = 1). In both previous phylogenetic analyses of Coccinellini, four main clades were recognized, but the phylogenetic relationships between them were inconsistent due to different molecular datasets used and sampling (Nattier et al. 2021, Tomaszewska et al. 2021). This study supports the relationship (Clade D + (Clade C + (Clade B + Clade A))), which is consistent with the phylogenetic reconstruction results of Nattier et al. (2021). However, it is different from the relationship (((Clade D + Clade C) + Clade B) + Clade A) proposed by Tomaszewska et al. (2021). Coccinellini was recovered as the sister group of Chilocorini with high support in our phylogenetic analyses (BS = 100, PP = 1). This is congruent with some previous studies (Magro et al. 2010; Seago et al. 2011; Robertson et al. 2015; Escalona et al. 2017; Tomaszewska et al. 2021). However, this result may be due to inadequate sampling in our and previous phylogenetic studies. Recently, the phylogeny of Coccinellidae based on extensive sampling placed the Coccinellini and either Sticholotidini (Che et al. 2021) or Plotonini (Li et al. 2019) as a sister group.

Within phytophagous ladybird beetles, the sister group between *Epiverta chelonia* and *Subcoccinella vigintiquattuorpunctata* was found and embedded within the Epilachnini in all our analyses. This situation rendered the Epilachnini to be nonmonophyletic, as previously reported in two phylogenetic analyses of Coccinellidae using mitogenomes (Song et al. 2020; Iqbal et al. 2024). Similar results were shown in an earlier study (Szawaryn et al. 2015), in which the evolutionary history of Epilachnini was inferred by four DNA markers (*18S rDNA*, *28S rDNA*, *lrRNA* and *COI*) and 104 morphological characters for 153 species representing all previously recognized genera. This study suggested that Epivertini and Subcoccinellini should be relegated to the genus of the tribe Epilachnini; thus, more evidence is required to better understand the phylogenetic relationships of this tribe.

Scymnini, usually small and dorsally pubescent, is a diverse and most species‐rich tribe in Coccinellidae. This tribe was recovered as a monophyletic clade in our study, regardless of datasets or phylogenetic inference approaches, which is different from those of previous studies (Magro et al. 2010; Robertson et al. 2015; Che et al. 2021). The different conclusions are mainly due to the inconsistent sample coverage of Scymnini between our study and previous studies. We also found the phylogenetic placements of *Coccidula rufa* and *Rodolia quadrimaculata* are sensitive to the choice of analysis approaches and datasets (Figure 6, Figure S4). This phenomenon supports our heterogeneity analysis that species with high heterogeneity tend to have unstable phylogenetic positions or incorrect grouping with uncorrelated clades (Guo et al. 2023). However, we observed that the phylogenetic placements of *Coccidula rufa* and *Rodolia quadrimaculata* are relatively more stable in ML analysis than those of BI (Figure 6 and Figure S4).

Overall, we phylogenetic results inferred from mitogenome sequences supported the monophyly of Coccinellini, Scymnini, and the phytophagous ladybird beetle clade. However, phylogenetic relationships among tribes within the Coccinellidae are still significantly confusing. The relatively small number of sequenced mitogenomes in ladybirds has prevented us from further drawing a conclusion about their phylogenetic relationships. Future studies should continue to complement the mitogenomes from unrepresented clades, such as the Sticholotidini, Stethorini, Cryptognathini, Aspidimerini, and Ortaliini, to gain a more comprehensive understanding of the phylogenetic relationships within Coccinellidae.

## Author Contributions

**Qiaoqiao Liu:** conceptualization (equal), data curation (equal), formal analysis (equal), methodology (equal), writing – original draft (equal), writing – review and editing (equal). **Pingzhou Zhu:** data curation (equal), investigation (equal). **Shiwen Xu:** formal analysis (equal), software (equal). **Chunyan Yang:** investigation (equal), methodology (equal). **Fan Song:** methodology (equal), software (equal), writing – review and editing (equal). **Yufang Meng:** investigation (equal). **Jinhong Zhou:** investigation (equal). **Hailin Yang:** funding acquisition (equal), project administration (equal), writing – review and editing (equal), writing – review and editing (equal). **Weidong Huang:** conceptualization (equal), project administration (equal), validation (equal), writing – original draft (equal), writing – review and editing (equal).

## Ethics Statement

The authors have nothing to report.

## Conflicts of Interest

The authors declare no conflicts of interest.

## Supporting information


Data S1.
**Table S1.** Sampling information of the mitogenome sequenced in this study.
**Table S2.** The taxa of the mitogenomes used to analyze in this study.
**Table S3.** List of annotated mitochondrial genes of *Calvia chinensis* and its characteristic features.
**Table S4.** List of annotated mitochondrial genes of *Harmonia eucharis* and its characteristic features.
**Table S5.** List of annotated mitochondrial genes of *Micraspis discolor* and its characteristic features.
**Table S6.** List of annotated mitochondrial genes of *Oenopia kirbyi* and its characteristic features.
**Table S7.** Base composition and skewness of the mitogenomes of *Calvia chinensis*, *Harmonia eucharis*, *Micraspis discolor* and *Oenopia kirbyi*.
**Figure S1.** The abnormal secondary structure of the tRNAs in the four newly sequenced mitogenome in this study.
**Figure S2.** AliGROOVE analysis of 66 Coccinellidae species based on PCGRNA dataset (A) and PCG12RNA dataset (B).
**Figure S3.** AliGROOVE analysis of 66 Coccinellidae species based on the first codon sit (A), second codon site (B) and third codon site (C).
**Figure S4.** Phylogenetic tree of Coccinellidae obtained from PhyloBayesian inference based on the datasets of PCGRNA and PCG12RNA.

## Data Availability

The annotated mitogenomic sequences of *Calvia chinensis*, *Harmonia eucharis*, *Micraspis discolor* , and *Oenopia kirby* have been deposited in GenBank (https://www.ncbi.nlm.nih.gov/) under the accession numbers PP926237–PP926240.
