# Supplementary material for: Comparative Mitogenomes and Phylogenetic Analyses of Coccinellidae (Coleoptera: Coccinelloidea)
Source: Ecol Evol. 2025 Mar 20;15(3):e71053. doi: 10.1002/ece3.71053 (PMC11925485; doi:10.1002/ece3.71053)
Supplement: Supplementary file 1 — Data S1. Table S1. Sampling information of the mitogenome sequenced in this study. Table S2. The taxa of the mitogenomes used to analyze in this study. Table S3. List of annotated mitochondrial genes of Calvia chinensis and its characteristic features. Table S4. List of annotated mitochondrial genes of Harmonia eucharis and its characteristic features. Table S5. List of annotated mitochondrial genes of Micraspis discolor and its characteristic features. Table S6. List of annotated mitochondrial genes of Oenopia kirbyi and its characteristic features. Table S7. Base composition and skewness of the mitogenomes of Calvia chinensis, Harmonia eucharis, Micraspis discolor and Oenopia kirbyi. Figure S1. The abnormal secondary structure of the tRNAs in the four newly sequenced mitogenome in this study. Figure S2. AliGROOVE analysis of 66 Coccinellidae species based on PCGRNA dataset (A) and PCG12RNA dataset (B). Figure S3. AliGROOVE analysis of 66 Coccinellidae species based on the first codon sit (A), second codon site (B) and third codon site (C). Figure S4. Phylogenetic tree of Coccinellidae obtained from PhyloBayesian inference based on the datasets of PCGRNA and PCG12RNA. [file ECE3-15-e71053-s001.docx]

**Appendix:**

**TABLE S1** Sampling information of the mitogenome sequenced in this study.

| Species | Location | Date | Longitude | Latitude | Altitude |
| --- | --- | --- | --- | --- | --- |
| *Calvia chinensis* | Zunyi, Guizhou, China | 2021/7/22 | 107.714° | 27.729° | 896 m |
| *Micraspis discolor* | Zunyi, Guizhou, China | 2021/7/22 | 107.714° | 27.729° | 896 m |
| *Harmonia eucharis* | Yis Hvib, Guizhou, China | 2021/7/19 | 105.128° | 25.3499° | 1521 m |
| *Oenopia kirbyi* | Yis Hvib, Guizhou, China | 2021/7/19 | 105.128° | 25.349° | 1521 m |

**TABLE S2** The taxa of the mitogenomes used to analyses in this study.

| **Family** | **Tribe** | **Species** | **GenBank number** | **References** |
| --- | --- | --- | --- | --- |
| Bothrideridae | Bothriderini | *Dastarcus helophoroides* | NC_024271 | Zhang et al. 2015 |
| Discolomatidae |  | Discolomatinae sp. | JX412748 | Unpublished |
| Endomychidae |  | *Sinocymbachus quadrimaculatus* | MT554390 | Unpublished |
|  |  | *Ancylopus pictus* | PP356720 | Unpublished |
| Latridiidae |  | *Cartodere nodifer* | OQ716327 | Unpublished |
| Coccinellidae | Coccidulini | *Coccidula rufa* | JX412767 | Unpublished |
|  |  | *Cryptolaemus montrouzieri* | KT874575 | Unpublished |
|  | Noviini | *Rodolia quadrimaculata* | MN053055 | Song et al. 2020 |
|  | Coccinellini | *Adalia bipunctata* | MW029465 | Li et al. 2021 |
|  |  | *Aiolocaria hexaspilota* | MK583344 | Seo et al. 2019 |
|  |  | *Anatis ocellata* | NC_036272 | Unpublished |
|  |  | *Anisosticta novemdecimpunctata* | KT876880 | Linard et al. 2016 |
|  |  | *Calvia championorum* | KX132085 | Unpublished |
|  |  | ***Calvia chinensis*** | **PP926237** | **This study** |
|  |  | *Calvia decemguttata* | KX087252 | Unpublished |
|  |  | *Calvia muiri* | MF992928 | Unpublished |
|  |  | *Cycloneda sanguinea* | KU877170 | Unpublished |
|  |  | *Coccinella lama* | MW029464 | Li et al. 2021 |
|  |  | *Coleomegilla maculata* | MZ303005 | Unpublished |
|  |  | *Cheilomenes sexmaculata* | MZ334467 | Unpublished |
|  |  | *Coccinella transversoguttata* | NC_067078 | Unpublished |
|  |  | *Coccinella septempunctata* | OU015583 | Unpublished |
|  |  | *Eriopis connexa* | MG253268 | Unpublished |
|  |  | *Eriopis patagonia* | MN509443 | Salazar & Nattier 2020 |
|  |  | *Harmonia axyridis* | MW029463 | Li et al. 2021 |
|  |  | ***Harmonia eucharis*** | **PP926238** | **This study** |
|  |  | *Harmonia quadripunctata* | KX087296 | Unpublished |
|  |  | *Hippodamia undecimnotata* | KX087298 | Unpublished |
|  |  | *Hippodamia convergens* | KX755331 | Unpublished |
|  |  | *Hippodamia variegata* | NC_046481 | Hao et al. 2019 |
|  |  | *Illeis cincta* | MF992929 | Unpublished |
|  |  | *Illeis bistigmosa* | NC_061950 | Unpublished |
|  |  | *Illeis koebelei* | NC_066052 | Unpublished |
|  |  | *Lemnia saucia* | MK574678 | Zhou et al. 2019 |
|  |  | *Megalocaria dilatate* | NC_064320 | Unpublished |
|  |  | ***Micraspis discolor*** | **PP926239** | **This study** |
|  |  | *Oenopia dracoguttata* | MW029467 | Li et al. 2021 |
|  |  | *Oenopia sauzeti* | NC_058236 | Unpublished |
|  |  | *Oenopia formosana* | NC_085345 | Unpublished |
|  |  | ***Oenopia kirbyi*** | **PP926240** | **This study** |
|  |  | *Olla v-nigrum* | MZ303015 | Unpublished |
|  |  | *Propylea quattuordecimpunctata* | MF992931 | Unpublished |
|  |  | *Propylea japonica* | KM244660 | Tang et al. 2014 |
|  |  | *Halyzia sedecimguttata* | KT780652 | Unpublished |
|  |  | *Halyziini* sp. | MG584728 | Unpublished |
|  |  | *Psyllobora lenta* | MZ303017 | Unpublished |
|  |  | *Vibidia duodecimguttata* | NC_066406 | Unpublished |
|  | Chilocorini | *Chilocorus bipustulatus* | MN053054 | Song et al. 2020 |
|  |  | *Chilocorus rubidus* | NC_082311 | Unpublished |
|  | Epilachnini | *Afissula kambaitana* | MF992930 | Unpublished |
|  |  | *Epilachna admirabilis* | MN053053 | Song et al. 2020 |
|  |  | *Afissula* sp. | MN053057 | Song et al. 2020 |
|  |  | *Henosepilachna* sp. | MT548778 | Unpublished |
|  |  | *Henosepilachna pusillanima* | NC_023469 | Behere et al. 2014 |
|  |  | *Henosepilachna vigintioctopunctata* | NC_041172 | Unpublished |
|  | Subcoccinellini | *Subcoccinella vigintiquattuorpunctata* | KT780695 | Unpublished |
|  | Epivertini | *Epiverta chelonia* | NC_064321 | Unpublished |
|  | Hyperaspidini | *Brachiacantha groendali* | MZ303003 | Unpublished |
|  |  | *Hyperaspis festiva* | MZ303012 | Unpublished |
|  | Scymnini | *Scymnu*s sp. | KT780638 | Unpublished |
|  |  | *Diomus seminulus* | MZ303008 | Unpublished |
|  |  | *Nephus includens* | MN164642 | Margo et al. 2019 |
|  |  | *Nephus reunion* | MN164643 | Margo et al. 2019 |
|  |  | *Nephus* *apolonia* | MN164644 | Margo et al. 2019 |
|  |  | *Nephus voeltzkowi* | MN164646 | Margo et al. 2019 |
|  |  | *Scymnus loewii* | MZ303019 | Unpublished |
|  |  | *Scymnus rubricaudus* | MZ303020 | Unpublished |
|  |  | *Nephus oblongosignatus* | MT445723 | Margo et al. 2020 |
|  |  | *Scymnus canariensis* | OQ716382 | Unpublished |
|  |  | *Scymnus* *cardi* | PP639204 | Unpublished |
|  | Microweiseini | *Coccidophilus cariba* | MN447521 | Nattier & Salazar 2019 |

**TABLE S3** List of annotated mitochondrial genes of *Calvia chinensis* and its characteristic features.

| Gene | Direction | Location | Size  (bp) | Anticodon | Codon  Start Stop | | Intergenic nucleotides |
| --- | --- | --- | --- | --- | --- | --- | --- |
| *trnI* | F | 1-65 | 65 | GAT |  |  | 0 |
| *trnQ* | R | 1593-1661 | 69 | TTG |  |  | 1527 |
| *trnM* | F | 1659-1726 | 68 | CAT |  |  | -3 |
| *ND2* | F | 1727-2732 | 1006 |  | ATT | T | 0 |
| *trnW* | F | 2733-2795 | 63 | TCA |  |  | 0 |
| *trnC* | R | 2788-2850 | 63 | GCA |  |  | -8 |
| *trnY* | R | 2853-2915 | 63 | GTA |  |  | 2 |
| *COI* | F | 2890-4450 | 1561 |  | ATC | T | -26 |
| *trnL* | F | 4451-4514 | 64 | TAA |  |  | 0 |
| *COII* | F | 4515-5193 | 679 |  | ATA | T | 0 |
| *trnK* | F | 5194-5263 | 70 | CTT |  |  | 0 |
| *trnD* | F | 5263-5328 | 66 | GTC |  |  | -1 |
| *ATP8* | F | 5329-5481 | 153 |  | ATC | TAG | 0 |
| *ATP6* | F | 5475-6132 | 658 |  | ATG | T | -7 |
| *COIII* | F | 6133-6913 | 781 |  | ATG | T | 0 |
| *trnG* | F | 6914-6975 | 62 | TCC |  |  | 0 |
| *ND3* | F | 6976-7327 | 352 |  | ATT | T | 0 |
| *trnA* | F | 7328-7390 | 63 | TGC |  |  | 0 |
| *trnR* | F | 7391-7454 | 64 | TCG |  |  | 0 |
| *trnN* | F | 7457-7520 | 64 | GTT |  |  | 2 |
| *trnS* | F | 7521-7576 | 56 | TCT |  |  | 0 |
| *trnE* | F | 7577-7639 | 63 | TTC |  |  | 0 |
| *trnF* | R | 7638-7701 | 64 | GAA |  |  | -2 |
| *ND5* | R | 7702-9415 | 1714 |  | ATT | T | 0 |
| *trnH* | R | 9416-9478 | 63 | GTG |  |  | 0 |
| *ND4* | R | 9479-10,799 | 1321 |  | ATG | T | 0 |
| *ND4L* | R | 10,793-11,071 | 279 |  | ATG | TAA | -7 |
| *trnT* | F | 11,072-11,136 | 65 | TGT |  |  | 0 |
| *trnP* | R | 11,137-11,197 | 61 | TGG |  |  | 0 |
| *ND6* | F | 11,212-11,692 | 481 |  | ATA | T | 14 |
| *CYTB* | F | 11,693-12,830 | 1138 |  | ATG | T | 0 |
| *trnS* | F | 12,831-12,893 | 63 | TGA |  |  | 0 |
| *ND1* | R | 12,910-13,851 | 942 |  | ATG | TAG | 16 |
| *trnL* | R | 13,852-13,913 | 62 | TAG |  |  | 0 |
| *lrRNA* | R | 13,914-15,204 | 1291 |  |  |  | 0 |
| *trnV* | R | 15,205-15,267 | 63 | TAC |  |  | 0 |
| *srRNA* | R | 15,268-16,092 | 825 | TTG |  |  | 0 |
| CR | F | 16,093-18,841 | 2749 | CAT |  |  | 0 |

**TABLE S4** List of annotated mitochondrial genes of *Harmonia eucharis* and its characteristic features.

| Gene | Direction | Location | Size  (bp) | Anticodon | Codon  Start Stop | | Intergenic nucleotides |
| --- | --- | --- | --- | --- | --- | --- | --- |
| *trnI* | F | 1-64 | 64 | GAT |  |  | 0 |
| *trnQ* | R | 903-971 | 69 | TTG |  |  | 838 |
| *trnM* | F | 969-1036 | 68 | CAT |  |  | -3 |
| *ND2* | F | 1037-2039 | 1003 |  | ATT | T | 0 |
| *trnW* | F | 2040-2102 | 63 | TCA |  |  | 0 |
| *trnC* | R | 2095-2156 | 62 | GCA |  |  | -8 |
| *trnY* | R | 2157-2220 | 64 | GTA |  |  | 0 |
| *COI* | F | <2222-3755 | >1534 |  | --- | T | 1 |
| *trnL* | F | 3756-3820 | 65 | TAA |  |  | 0 |
| *COII* | F | 3821-4493 | 673 |  | ATA | T | 0 |
| *trnK* | F | 4494-4564 | 71 | CTT |  |  | 0 |
| *trnD* | F | 4564-4626 | 63 | GTC |  |  | -1 |
| *ATP8* | F | 4627-4779 | 153 |  | ATC | TAA | 0 |
| *ATP6* | F | 4773-5430 | 658 |  | ATG | T | -7 |
| *COIII* | F | 5431-6211 | 781 |  | ATG | T | 0 |
| *trnG* | F | 6212-6273 | 62 | TCC |  |  | 0 |
| *ND3* | F | 6274-6625 | 352 |  | ATC | T | 0 |
| *trnA* | F | 6626-6687 | 62 | TGC |  |  | 0 |
| *trnR* | F | 6687-6749 | 63 | TCG |  |  | -1 |
| *trnN* | F | 6747-6811 | 65 | GTT |  |  | -3 |
| *trnS* | F | 6812-6867 | 56 | TCT |  |  | 0 |
| *trnE* | F | 6868-6930 | 63 | TTC |  |  | 0 |
| *trnF* | R | 6929-6993 | 65 | GAA |  |  | -1 |
| *ND5* | R | 6994-8707 | 1714 |  | ATT | T | 0 |
| *trnH* | R | 8708-8770 | 63 | GTG |  |  | 0 |
| *ND4* | R | 8771-10,091 | 1321 |  | ATG | T | 0 |
| *ND4L* | R | 10,085-10,363 | 279 |  | ATG | TAA | -7 |
| *trnT* | F | 10,364-10,428 | 65 | TGT |  |  | 0 |
| *trnP* | R | 10,429-10,489 | 61 | TGG |  |  | 0 |
| *ND6* | F | 10,504-10,984 | 481 |  | ATA | T | 14 |
| *CYTB* | F | 10,985-12,122 | 1138 |  | ATG | T | 0 |
| *trnS* | F | 12,123-12,187 | 65 | TGA |  |  | 0 |
| *ND1* | R | 12,205-13,146 | 942 |  | ATT | TAG | 17 |
| *trnL* | R | 13,147-13,208 | 62 | TAG |  |  | 0 |
| *lrRNA* | R | 13,209-14,486 | 1278 |  |  |  | 0 |
| *trnV* | R | 14,487-14,549 | 63 | TAC |  |  | 0 |
| *srRNA* | R | 14,550-15,356 | 807 |  |  |  | 0 |
| CR | F | 15,357-17,133 | 1777 |  |  |  | 0 |

Note: < indicates that PCG lacks a start codon.

**TABLE S5** List of annotated mitochondrial genes of *Micraspis discolor* and its characteristic features.

| Gene | Direction | | Location | Size  (bp) | Anticodon | Codon  Start Stop | | Intergenic nucleotides |
| --- | --- | --- | --- | --- | --- | --- | --- | --- |
| *trnI* | | F | 1-63 | 63 | GAT |  |  | 0 |
| *trnQ* | | R | 1560-1628 | 69 | TTG |  |  | 1496 |
| *trnM* | | F | 1626-1693 | 68 | CAT |  |  | -3 |
| *ND2* | | F | 1694-2699 | 1006 |  | ATT | T | 0 |
| *trnW* | | F | 2700-2762 | 63 | TCA |  |  | 0 |
| *trnC* | | R | 2755-2814 | 60 | GCA |  |  | -8 |
| *trnY* | | R | 2815-2876 | 62 | GTA |  |  | 0 |
| *COI* | | F | <2878-4411 | >1534 |  | --- | T | 1 |
| *trnL* | | F | 4412-4474 | 63 | TAA |  |  | 0 |
| *COII* | | F | 4475-5153 | 679 |  | ATT | T | 0 |
| *trnK* | | F | 5154-5223 | 70 | CTT |  |  | 0 |
| *trnD* | | F | 5224-5287 | 64 | GTC |  |  | -1 |
| *ATP8* | | F | 5288-5440 | 153 |  | ATA | TAA | 0 |
| *ATP6* | | F | 5434-6090 | 657 |  | ATG | T | -7 |
| *COIII* | | F | 6091-6871 | 781 |  | ATG | T | 0 |
| *trnG* | | F | 6872-6933 | 62 | TCC |  |  | 0 |
| *ND3* | | F | 6934-7285 | 352 |  | ATA | T | 0 |
| *trnA* | | F | 7286-7347 | 62 | TGC |  |  | 0 |
| *trnR* | | F | 7347-7407 | 61 | TCG |  |  | -1 |
| *trnN* | | F | 7405-7467 | 63 | GTT |  |  | -3 |
| *trnS* | | F | 7468-7521 | 54 | TCT |  |  | 0 |
| *trnE* | | F | 7522-7583 | 62 | TTC |  |  | 0 |
| *trnF* | | R | 7637-7699 | 63 | GAA |  |  | 53 |
| *ND5* | | R | 7700-9407 | 1708 |  | ATT | T | 0 |
| *trnH* | | R | 9408-9469 | 62 | GTG |  |  | 0 |
| *ND4* | | R | 9470-107,90 | 1321 |  | ATG | T | 0 |
| *ND4L* | | R | 10,784-11,062 | 279 |  | ATG | TAA | -7 |
| *trnT* | | F | 11,063-11,125 | 63 | TGT |  |  | 0 |
| *trnP* | | R | 11,126-11,186 | 61 | TGG |  |  | 0 |
| *ND6* | | F | 11,189-11,663 | 475 |  | ATC | T | 2 |
| *CYTB* | | F | 11,664-12,801 | 1138 |  | ATG | T | 0 |
| *trnS* | | F | 12,802-12,863 | 62 | TGA |  |  | 0 |
| *ND1* | | R | 12,881-13,825 | 945 |  | ATA | TAG | 17 |
| *trnL* | | R | 13,826-13,886 | 61 | TAG |  |  | 0 |
| *lrRNA* | | R | 13,887-15,172 | 1286 |  |  |  | 0 |
| *trnV* | | R | 15,173-15,233 | 61 | TAC |  |  | 0 |
| *srRNA* | | R | 15,234-16,047 | 814 | TTG |  |  | 0 |
| CR | | F | 16,048-17,508 | 1461 | CAT |  |  | 0 |

Note: < indicates that PCG lacks a start codon.

**TABLE S6.** List of annotated mitochondrial genes of *Oenopia kirbyi* and its characteristic features.

| Gene | Direction | | Location | Size  (bp) | Anticodon | Codon  Start Stop | | Intergenic nucleotides |
| --- | --- | --- | --- | --- | --- | --- | --- | --- |
| *trnI* | | F | 1-63 | 63 | GAT |  |  | 0 |
| *trnQ* | | R | 3399-3467 | 69 | TTG |  |  | 3335 |
| *trnM* | | F | 3465-3532 | 68 | CAT |  |  | -3 |
| *ND2* | | F | 3533-4538 | 1006 |  | ATC | T | 0 |
| *trnW* | | F | 4539-4601 | 63 | TCA |  |  | 0 |
| *trnC* | | R | 4594-4655 | 62 | GCA |  |  | -8 |
| *trnY* | | R | 4656-4718 | 63 | GTA |  |  | 0 |
| *COI* | | F | 4693-6253 | 1561 |  | ATC | T | -26 |
| *trnL* | | F | 6254-6316 | 63 | TAA |  |  | 0 |
| *COII* | | F | 6317-6995 | 679 |  | ATA | T | 0 |
| *trnK* | | F | 6996-7064 | 69 | CTT |  |  | 0 |
| *trnD* | | F | 7065-7131 | 67 | GTC |  |  | 0 |
| *ATP8* | | F | 7132-7284 | 153 |  | ATT | TAA | 0 |
| *ATP6* | | F | 7278-7934 | 657 |  | ATG | T | -7 |
| *COIII* | | F | 7936-8716 | 781 |  | ATG | T | 0 |
| *trnG* | | F | 8717-8778 | 62 | TCC |  |  | 0 |
| *ND3* | | F | 8779-9130 | 352 |  | ATT | T | 0 |
| *trnA* | | F | 9131-9193 | 63 | TGC |  |  | 0 |
| *trnR* | | F | 9193-9257 | 65 | TCG |  |  | -1 |
| *trnN* | | F | 9255-9318 | 64 | GTT |  |  | -3 |
| *trnS* | | F | 9319-9374 | 56 | TCT |  |  | 0 |
| *trnE* | | F | 9375-9437 | 63 | TTC |  |  | 0 |
| *trnF* | | R | 9436-9498 | 63 | GAA |  |  | -2 |
| *ND5* | | R | 9499-11,194 | 1696 |  | ATT | T | 0 |
| *trnH* | | R | 11,210-11,273 | 64 | GTG |  |  | 15 |
| *ND4* | | R | 11,274-12,594 | 1321 |  | ATG | T | 0 |
| *ND4L* | | R | 12,588-12,863 | 276 |  | ATG | TAA | -7 |
| *trnT* | | F | 12,864-12,928 | 65 | TGT |  |  | 0 |
| *trnP* | | R | 12,929-12,989 | 61 | TGG |  |  | 0 |
| *ND6* | | F | 12,992-13,475 | 484 |  | ATT | T | 2 |
| *CYTB* | | F | 13,476-14,614 | 1139 |  | ATG | T | 0 |
| *trnS* | | F | 14,615-14,679 | 65 | TGA |  |  | 0 |
| *ND1* | | R | 14,697-15,629 | 933 |  | ATA | TAG | 17 |
| *trnL* | | R | 15,642-15,703 | 62 | TAG |  |  | 0 |
| *lrRNA* | | R | 15,704-17,002 | 1299 |  |  |  | 0 |
| *trnV* | | R | 17,003-17,068 | 66 | TAC |  |  | 0 |
| *srRNA* | | R | 17,069-17,897 | 829 | TTG |  |  | 0 |
| CR | | F | 17,899-19,634 | 1736 | CAT |  |  | 0 |

**TABLE S7** Base composition and skewness of the mitogenomes of *Calvia chinensis*, *Harmonia eucharis*, *Micraspis discolor* and *Oenopia kirbyi*.

| Feature | A + T % | AT-skew | GC-skew |
| --- | --- | --- | --- |
| Mitogenome | 79.03/76.10/78.79/79.05 | 0.056/0.050/0.050/0.026 | -0.126/-0.227/-0.189/-0.146 |
| PCGs | 77.43/74.85/77.21/77.91 | -0.146/-0.139/-0.142/-0.138 | 0.008/-0.021/0.008/-0.008 |
| tRNAs | 78.35/76.64/79.33/78.95 | 0.035/0.024/0.015/0.014 | 0.171/0.134/0.137/0.135 |
| rRNAs | 82.56/80.24/81.67/81.91 | -0.082/-0.058/-0.107/-0.059 | 0.317/0.374/0.330/0.335 |
| CR | 85.59/79.80/85.56/83.93 | 0.082/0.014/0.042/-0.010 | -0.232/-0.287/-0.156/-0.219 |

Note: the data separated by “/” correspond to the four mitogenomes in turn of *Calvia chinensis*, *Harmonia eucharis*, *Micraspis discolor* and *Oenopia kirbyi*.

**FIGURE S1** The abnormal (lack of TψC arm or DHU arm) secondary structure of the tRNAs in the four newly sequenced mitogenome in this study.

**FIGURE S2** AliGROOVE analysis of 66 Coccinellidae species based on PCGRNA dataset (**A**) and PCG12RNA dataset (B). The mean similarity score between sequences is represented by colored squares, based on AliGROOVE scores ranging from *-*1, which indicates a great difference in rates from the remainder of the data set, that is, heterogeneity (red coloring), to +1, which indicates rates that matched all other comparisons (blue coloring).

**FIGURE S3** AliGROOVE analysis of 66 Coccinellidae species based on the first codon sit (**A**), second codon site (B) and third codon site (C). The mean similarity score between sequences is represented by colored squares, based on AliGROOVE scores ranging from *-*1, which indicates a great difference in rates from the remainder of the data set, that is, heterogeneity (red coloring), to +1, which indicates rates that matched all other comparisons (blue coloring).

**FIGURE S4** Phylogenetic tree of Coccinellidae obtained from PhyloBayesian inference based on the datasets of PCGRNA and PCG12RNA. Different tribes are highlighted by colors. The red spot in the node of tree indicated the dispute between two trees. Posterior probabilities values are shown near the nodes.

**References**

Zhang, Z., Wang, X., Li, R., Guo, R., Zhang, W., Song, W., Hao, C., Wang, H., & Li, M. (2015). The mitochondrial genome of *Dastarcus helophoroides* (Coleoptera: Bothrideridae) and related phylogenetic analyses. *Gene*, 560, 15–24.

Song, N., Li, X., Yin, X., Li, X., & Xi, Y. (2020). The mitochondrial genomes of ladybird beetles and implications for evolution and phylogeny. *International Journal of Biological Macromolecules*, 147, 1193–1203.

Li, X., Song, N., & Zhang, H. (2021). Comparative and phylogenomic analyses of mitochondrial genomes in Coccinellidae (Coleoptera: Coccinelloidea). *PeerJ*, 9, e12169.

Seo, B.Y., Park, J., Kwon, W., & Park, J. (2019). The complete mitochondrial genome of *Aiolocaria hexaspilota* (Hope, 1831) (Coleoptera:Coccinellidae). *Mitochondrial DNA Part B-Resources*, 4, 1472-1474.

Linard, B., Arribas, P., Andújar, C., Crampton‐Platt, A., & Vogler, A.P. (2016). Lessons from genome skimming of arthropod‐preserving ethanol. *Molecular Ecology Resources*, 16, 1365-1377.

Salazar, K., and Nattier, R. (2020). New light on historical specimens reveals a new species of ladybird (Coleoptera: Coccinellidae): morphological, museomic, and phylogenetic analyses. *Insects*, 11, 766.

Hao, Y.N., Liu, C.Z., & Sun, Y.X. (2019). The complete mitochondrial genome of the Adonis ladybird, *Hippodamia variegata* (Coleoptera: Coccinellidae). *Mitochondrial DNA Part B-Resources*, 4, 1087–1088.

Zhou, J., Li, R., Kong, Y., Hu, H.-W., and Shu, X.-H. (2019). Mitochondrial genome of *Lemnia saucia* Mulsant (Coleoptera: Coccinellidae) and phylogenetic analysis. *Mitochondrial DNA Part B-Resources*, 4, 1441–1442.

Tang, M., Tan, M., Meng, G., Yang, S., Su, X., Liu, S., Song, W., Li, Y., Wu, Q., Zhang, A., & Zhou, X. (2014). Multiplex sequencing of pooled mitochondrial genomes—a crucial step toward biodiversity analysis using mito-metagenomics. *Nucleic Acids Research*, 42, e166.

Behere, G.T., Firake, D.M., Tay, W.T., Azad Thakur, N.S., & Ngachan, S.V. (2014). Complete mitochondrial genome sequence of a phytophagous ladybird beetle, *Henosepilachna pusillanima* (Mulsant) (Coleoptera: Coccinellidae). *Mitochondrial DNA Part A*, 27, 291–292.

Magro, A., Lecompte, E., Hemptinne, J.L., Soares, A.O., Dutrillaux, A.M., Murienne, J., Fürsch, H., & Dutrillaux, B. (2019). First case of parthenogenesis in ladybirds (Coleoptera: Coccinellidae) suggests new mechanisms for the evolution of asexual reproduction. *Journal of Zoological Systematics and Evolutionary Research*, 58, 194–208.

Magro, A., Churata-Salcedo, J., Lecompte, E., Hemptinne, J.L., & Almeida, L.M. (2020). A new species of *Nephus* (*Nephus*) (Coleoptera, Coccinellidae) described from Reunion Island. *ZooKeys* 962, 123–137.

Nattier, R., & Salazar, K. (2019). Next-generation sequencing yields mitochondrial genome of Coccidophilus cariba Gordon (Coleoptera: Coccinellidae) from museum specimen. *Mitochondrial DNA Part B-Resources*, 4, 3780–3781.
